# Shape Variation in the Craniomandibular System and Prevalence of Dental Problems in Domestic Rabbits: A Case Study in Evolutionary Veterinary Science

**DOI:** 10.3390/vetsci4010005

**Published:** 2017-01-24

**Authors:** Christine Böhmer, Estella Böhmer

**Affiliations:** 1 UMR 7179 CNRS, Muséum National d’Histoire Naturelle, CP 55, 57 rue Cuvier, 75231 Paris Cedex 05, France; 2 Chirurgische und Gynäkologische Kleintierklinik ,Tierärztliche Fakultät, Ludwig-Maximilians-Universität München, Veterinärstr 13, München 80539, Germany; e.boehmer@lmu.de

**Keywords:** masticatory apparatus, axial load, malocclusion, reference lines, Lagomorpha, evolutionary morphology, phenotypic plasticity

## Abstract

In contrast to wild lagomorphs, pet rabbits exhibit a noticeably high frequency of dental problems. Although dietary habits are considered as a major factor contributing to acquired malocclusions, the exact causes and interrelationships are still under debate. In this regard, an important aspect that has not been considered thoroughly to date is the effect of diet-induced phenotypic plasticity in skull morphology. Therefore, we conducted a geometric morphometric analysis on skull radiological images of wild and pet rabbits in order to quantify intraspecific variation in craniomandibular morphology. The statistical analyses reveal a significant morphological differentiation of the craniomandibular system between both groups. Furthermore, the analysis of covariance shows that the force-generating modules (cranium and mandible) vary independently from the force-receiving module (hypselodont teeth) in pet rabbits, which is in contrast to their wild relatives. Our findings suggest that the phenotypic changes in domestic rabbits impact mastication performance and, consequently, oral health. An adequate close-to-nature nutrition throughout the whole life and especially beginning early parallel to weaning (phase of increased phenotypic plasticity) is necessary to ensure a normal strain on the teeth by promoting physiological lateral gliding movements and avoiding direct axial loads.

## 1. Introduction

All breeds of domestic rabbits descend from the European rabbit *Oryctolagus cuniculus*, which is a member of the family Leporidae (rabbits and hares). A remarkable peculiarity in veterinary medicine is the prevalence of dental problems among small herbivorous pet animals in general and rabbits in particular [1,2,3]. In pet rabbits, almost 90% of reported patients suffer from malocclusion caused by pathological tooth changes [1,4,5], in contrast to previous surveys that under-reported the frequency of dental problems: 30% [6], 38% [2]. Since obvious clinical evidence typically appears fairly late in the course of the dental disease, dental radiology is crucial for proper diagnosis [1,7,8,9]. Species-specific reference lines superimposed on radiographs enable objective interpretation of malocclusion in small pet animals [1,4,10,11]. Despite its proven usefulness in most domestic rabbits with a malocclusion, the anatomical reference lines appear to be not suitable for use in wild rabbits. This indicates intraspecific variation in skull morphology of the European rabbit (*Oryctolagus cuniculus*) and requires exploration in order to quantify morphological trends among domestic and wild rabbits.

**Figure 1:**
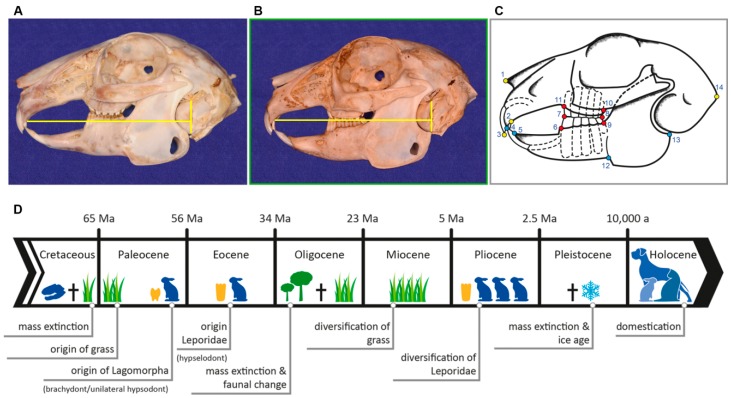
Morphology and evolution. (**A**) Craniomandibular morphology at occlusal resting pose in wild rabbit and (**B**) domestic rabbit in lateral view (scaled to same height). In pet rabbits without pathological changes of the skull or teeth, the species-specific reference line (yellow) begins at the rostral end of the hard palate immediately caudal to the second incisor and extends caudally to pass through the tympanic bulla at approximately one-third of its height (according to [4]); (**C**) Schematic representation of the 2D landmark set used in the present study (refer to Table 1 for description of landmarks). The color coding indicates the three landmark sub-sets representing the distinct modules: cranium (yellow), mandible (blue) and cheek teeth (red); (**D**) Simplified timeline of major evolutionary events concerning the origin of lagomorphs.

It is well known that the evolution of wild species into domestic forms by artificial selection has resulted in changes in behavior and morphology [12,13] and various fields of research have improved our understanding of animal domestication [14,15]. Recent molecular approaches offered insights into the genotypic variation underpinning morphological and behavioral traits e.g., [16,17,18,19]. However, as of yet, the genetic changes associated with animal domestication are still not fully understood because most domestic animals are genetically diverse and there are multiple genetic pathways producing domestic traits [20,21,22]. Comparative morphometric analyses revealed variation in the morphology of wild and domestic animals and related it to allometry and differences in the timing of developmental processes i.e., heterochrony) e.g., [13,23,24,25,26]. Most obvious morphological changes under domestication can be seen in the skull, but it appears that not all cranial areas vary equally (modularity) [27,28,29]. For instance, a modular separation of the nasomaxillary complex versus the neurocranium has been noted in the skull of canids [27,30]. A similar pattern of modularity has been reported for the skull of felids and the functional implications have been related to respiratory dysfunction [29]. In rabbits, domestication has resulted in changes in morphology as well, notably in skull shape. However, both domestic and wild rabbits have continuously growing, high-crowned teeth (hypselodont) and, thus, dentition is not basically affected by the domestication process (Figure 1A,B). This indicates at least two distinct modules in the cranium of *O. cuniculus*. Yet, the covariation among distinct modules in the skull of rabbits and the link between morphological variation and functional implications (in particular mastication) remains largely unknown.

A major factor contributing to the alarming situation with regard to dental problems in pet rabbits is the feeding behavior [31,32], which strongly depends on the human pet owner and mostly differs from the natural diet of their wild relatives [31]. However, from an evolutionary perspective, teeth are an adaptation to feeding [33,34,35,36], and since a dynamic relation exists between diet, the masticatory apparatus and oral health, it is reasonable that differences in diet affect the dentition and may be causative for pathological tooth changes. The skeleton and teeth of domestic rabbits reflect their phylogenetic heritage and, thus, it is important to consider the evolutionary history of *O. cuniculus* in order to understand the significant role of the highly specialized masticatory apparatus in rabbits including their hypselodont teeth.

The evolutionary history of Leporidae dates back to the Eocene [37] and is linked to the global tectonic and environmental changes during the Cenozoic Era [38] (Figure 1D). Originating from Asia, the radiation of leporids across Europe and North America began in particular after the major turnover in terrestrial ecosystems at the Eocene-Oligocene boundary (about 34 Ma) [39]. During the Eocene-Oligocene transition (EOT), a change from warm-humid forests with large-sized perissodactyls as dominant mammals to dry-temperature forest-steppe with open grasslands and with small rodents and lagomorphs as the dominant group occurred [39]. Leporids reached the highest diversity during the Pliocene (5−2.5 Ma) expanding to Africa and South America [38,40] and by this time the oldest evidence of the European rabbit genus *Oryctolagus* is noted in the fossil record, associated with an arid, warm savannah-type fauna [40]. The global cooling during the Pleistocene (2–5 Ma-10,000 a) caused a severe decline in leporid diversity and many genera became extinct [40]. *Oryctolagus* persists until today, but with only one species (*O. cuniculus*) left, and the last glacial period confined it to the Iberian Peninsula and southern France [40]. About 1500 years ago, rabbit domestication was initiated [14,41] and historical records as well as genetic evidence revealed a single origin of domestication in wild populations from France [42].

Due to the interaction between climate and vegetation, the global climate change in the early Cenozoic affected the evolution of the herbivorous rabbits. In this context, a main factor limiting the life span of mammals in the wild is tooth abrasion e.g., [43]. Hypselodont teeth compensate for intensive abrasion (resulting in loss of dental tissue) during food intake and food processing and are regarded as an evolutionary adaptation to the high abrasiveness of plants which is a consequence of an increased silica content (intrinsic by phytoliths in grasses, extrinsic by dust ingested with grass) [33,34,44,45]. Thus, it can be expected that a less abrasive diet may cause tooth overgrowth terminating in malocclusion. However, there is evidence showing that hypselodonty is accompanied by a regulatory mechanism for tooth growth compensating for differences in dental abrasion [46,47,48]. This topic is still under debate and although the dentition and skull form a functional unit, the specific effects of skull shape in domestic rabbits in this regard have been neglected to date.

Despite its importance for veterinary medicine, it is surprising to note that we still lack knowledge about the patterns of morphological changes in the skull and dentition of a number of domestic animals including rabbits. Here, we quantify skull morphology in *O. cuniculus* in order to address the following questions: (1) how does the entire skull morphology vary between wild and domestic rabbits; (2) to what extent is morphological variation in the skull modular and (3) what are the implications for the masticatory apparatus of domestic rabbits. Ultimately, we seek to provide an explanation for the high abundance of dental problems in pet rabbits from an evolutionary perspective. This will not only improve our understanding of the relation between morphology and pathologies in domestic animals, but is also an important case study of Evolutionary Veterinary Science (EvoVetSci).

## 2. Materials and Methods

### 2.1. Data Set and Radiographic Screening

In this case study, we examine the skulls of 12 mature European wild rabbits and 12 mature domestic rabbits belonging to the species *O. cuniculus*. The wild rabbits originate from southern Germany and Austria (victims of traffic accidents). The sex is unknown. Due to the aforementioned prevalence of dental problems among domestic rabbits (almost 90%), the sample is somewhat limited, but sufficient to yield a reasonable signal. The pet rabbit sample represents no specific breed with erect ears in order to avoid extreme phenotypes characteristic of certain breeds. The specimens are radiographed for medical reasons and not for the purpose of this study. Only specimens with adult dentition and without pathologies are included. Skull radiographs (laterolateral view) of anesthetized specimens are obtained with the mouth closed or open about one millimeter [4].

### 2.2. Shape Analysis

Landmark-based geometric morphometrics was used to quantify skull morphology and to analyze phenotypic differences. A total of 14 landmarks (LMs) in two dimensions were taken on laterolateral radiological images of the skull (Table 1, Figure 1C). The homologous osteological points were chosen in order to describe the skull morphology considering the species-specific reference lines by Böhmer and Crossley [4] that enable objective interpretation of malocclusion in small pet animals. Sets of landmarks were subsequently separated into three distinct modules: cranium (LM 1, 2, 3 and 14), mandible (LM 4, 5, 12 and 13), and cheek teeth (LM 6, 7, 8, 9, 10 and 11) (Figure 1C). Using the software tpsDig2 [49], type I and type II landmarks (sensu [50]) were digitized onto the skulls in lateral view. The digitalization of the landmarks was performed by a single author (EB) in order to prevent inter-observer measurement errors. The placement of the landmarks were repeated three times for each individual. The assessment of intra-observer variance revealed that the error is low ensuring reproducibility of the measurements. In order to superimpose geometries and to isolate size and shape, a generalized Procrustes analysis (GPA) was conducted using the software Morphologika² [51]. Next, a relative warp (RW) analysis was performed in the same software. The RW analysis summarized the multi-dimensional information and constructed a morphospace in which shape variation can be quantified. With the applied settings, this method is equivalent to a principal components analysis. The shape differences were visualized with thin-plate splines.

All subsequent analyses and statistical tests were performed using the software PAST [52]. In order to test if shape variation is a function of size, a multivariate regression analysis (log centroid size against RWs) was performed. Log transformed centroid size, the sum of squared distances of each landmark from the centroid of the skull, was used as a measure of size and RWs as a measure of shape [53]. A discriminant analysis was performed in order to test for significance of differences between the shapes of wild and domestic rabbits. Therefore, the RWs were subjected to a Hotelling’s T² test. The Procrustes distances of both groups (wild vs. domestic rabbits) from group mean shape were calculated as a measure of disparity (i.e., morphological diversity) [54].

### 2.3. Integration and Modularity

Modular covariation of the shape between cranium, mandible and cheek teeth is investigated using two-block partial least squares (2-block PLS) analysis [55] in the software PAST [52]. The program analyzes two blocks of shape data as separate configurations. Therefore, the data from the single Procrustes fit for the entire structure (skull) is divided into three subsets with equal number of landmark coordinates in order to analyze the modules against each other: module 1 (cranium) against module 2 (mandible), module 1 against module 3 (cheek teeth), and module 2 against module 3.

## 3. Results

The collected 2D LM coordinates of the analyzed specimens are available in supplementary Table S1.

### 3.1. Patterns of Morphological Diversification

**Figure 2:**
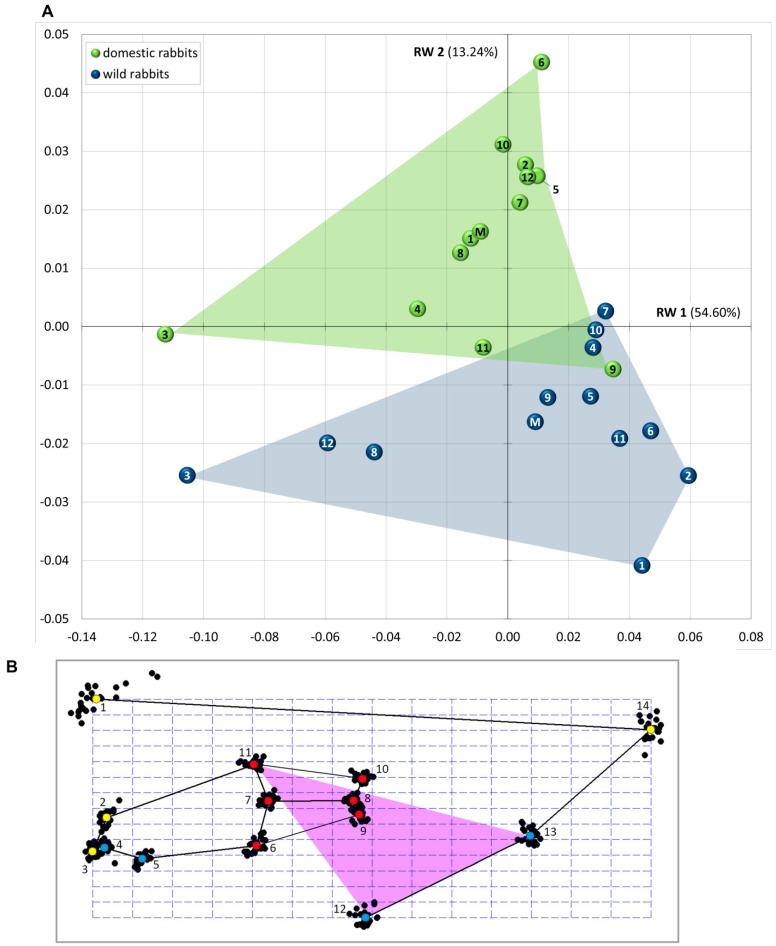

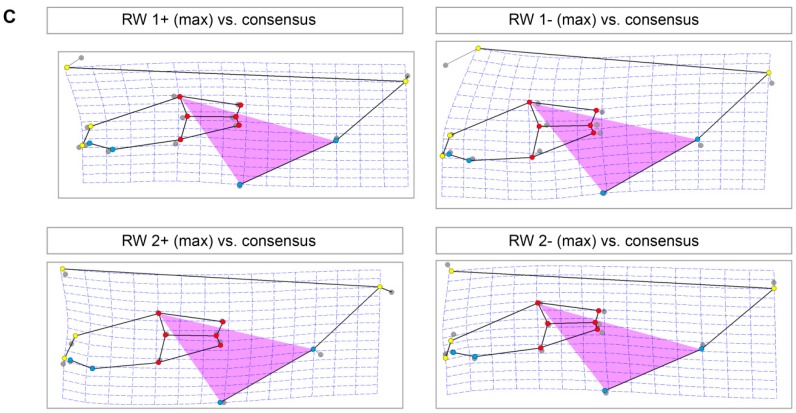
Relative warp (RW) analysis results. (**A**) The plot shows the morphological diversification of domestic and wild rabbits along RW 1 and 2. M = group mean configuration; (**B**) Scatterplot of all landmark configurations (black dots) and consensus shape (colored dots) as reference form after Procrustes superimposition. The purple area depicts the superficial masseter muscle (**C**) Thin-plate splines visualize the variation. The landmark configuration in grey represents the consensus shape (zero point in (**A**); equals mean shape of the sample as reference). The landmark configuration in color linked with black lines gives the shape information of the target shape associated with maximum and minimum RW scores, respectively.

About 95% of the total variance in the sample is explained by the first nine RWs (Table 2). The first three RWs account for more than 75% of the total variance in the sample (RW 1: 56.60%, RW 2: 13.24%, RW 3: 9.91%) and the morphospace constructed from RW 1 and RW 2 provides a reasonable approximation of the shape variation (Figure 2A). Wild and domestic rabbits are clearly separated along RW 2 and to a lesser extent along RW 1. The scatter plot reveals that the majority of wild rabbits fall in quadrant four, whereas almost all domestic rabbits lie in quadrant one and two, respectively.

**Figure 3:**
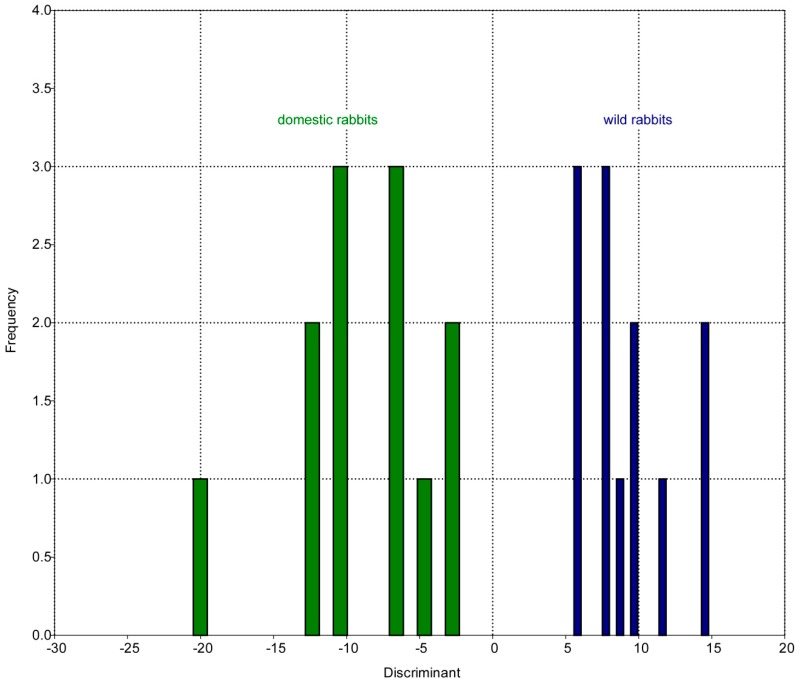
Discrimination analysis results. The histogram displays distinct separation between both groups of rabbits on basis of the morphological analysis.

The Hotelling’s T² test on the first nine RWs reveals that the mean difference values are significantly high (*p*-value < 0.001), predicting major differences between both groups. Figure 3 shows the result of the discrimination analysis. There is no significant difference between the morphological disparity (MD) of both groups: MD (wild rabbits) = 0.46 and MD (domestic rabbits) = 0.40 (*t*-test: *p*-value > 0.05).

In total, the geometric morphometric analysis thus indicates a distinct morphological differentiation between wild and domestic rabbit skulls.

### 3.2. Allometry: Size and Shape

The variance of log centroid size in wild and domestic rabbits is significantly different (*p* < 0.05). Means of log centroid size do not differ significantly between both groups (*p* > 0.05). Thus, the skull of pet rabbits reveals a larger variation in size than their wild counterparts. However, the average size is similar in both groups.

**Figure 4:**
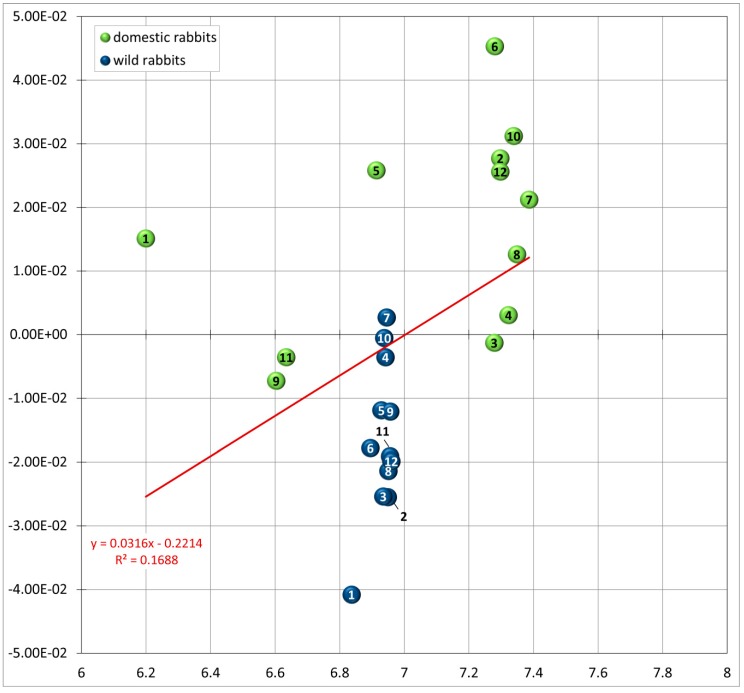
Regression of log centroid size (a measure of size) against the relative warp (RW) 2 (a measure of shape).

Allometry of the skull accounts partly for a portion of shape variation among rabbits. The multivariate regression of the shape variables (RW 1–9) on log centroid size shows that shape variation associated with RW 1, 3 and 5 to 9 are not a function of size (Table 3). There is an allometric relationship with RW 2 and RW 4 (*p* < 0.05). The amount of shape variation accounted for by the regressions is about 16.88% and 51.10%, respectively (Table 3). The plot of RW 2 against log centroid size reveals a separation of wild rabbits associated with negative RW 2 values and smaller log centroid size and domestic rabbits related with positive RW 2 values and larger log centroid size (Figure 4). The latter vary slightly in size, whereas wild rabbits are all of same size (Figure 4).

### 3.3. Landmark Variance

Comparing the consensus shape (mean shape of the sample) with all landmark configurations after Procrustes superimposition visualizes the shape variation within the sample (Figure 2B). It indicates the difference in location of corresponding landmarks. LM 1 (tip of nasal bone) reveals the highest variance by far (Table 4). Although by a magnitude smaller, LM 14 (tip of occipital protuberance) displays the second highest variance. The least variable homologous point is LM 3 (tip of incisor). LM 8 and 10 that characterize the last molar (m3), reveal a relatively low variance as well (Table 4).

### 3.4. Skull Shape Variation (Thin-Plate Splines)

RW 2 and to a lesser extent RW 1 separate wild and domestic rabbits. RW 1 and RW 2 primarily contrast anteroposteriorly elongated and dorsoventrally compressed skulls (positive RW 1 values, negative RW 2 values) against those that are anteroposteriorly compressed and dorsoventrally expanded (negative RW 1 values, positive RW 2 values) (Figure 2C). This pattern is driven in general by an overall change of the skull, but in particular combined by a change in the area of the nasal bone. Positive RW 1 and negative RW 2 scores are largely occupied by wild rabbits indicating that they tend to have a relatively long skull with the nasal bone projecting anteriorly over the incisors (Figure 2A,C). In contrast, negative RW 1 and positive RW 2 scores tend to characterize domestic rabbits displaying a relatively short skull and the tip of the nasal bone posterior to the incisors (Figure 2A,C). Other significant differences between both groups include the spatial displacement of important muscle unit attachment points, such as the relative position of the occipital protuberance and the angular process. In lateral view, the antegonial notch of the mandible lies on a vertical line with the last molars in wild rabbits (positive RW 1 values), whereas it is positioned posteriorly relative to the last molars in domestic rabbits (negative RW 1 values).

Focusing on RW 2, skull shape changes associated with positive RW 2 values include a shortening of the occipital region in anterior direction and a slight shift of the anterior cranial region (nasal bone and maxillary incisors) in dorsal direction (Figure 2C). Negative RW 2 values account for a shortening of the molar region in anterior direction and a slight shift of the anterior cranial region (nasal bone and maxillary incisors) in ventral direction. This pattern opposes domestic rabbits (positive RW 2 scores) against wild rabbits (negative RW 2 scores). In lateral view, the occiput is almost at the same level with the nasal bone in wild rabbits (negative RW 2 values), whereas it lies distinctly ventral to the tip of the nose in domestic rabbits (positive RW 2 values) (Figure 2C). Associated with positive RW 2 scores, the angular process is more or less at the same level with the tip of the lower incisors in wild rabbits. In contrast, associated with negative RW 2 scores, the angular process lies distinctly dorsal to the tip of the lower incisors in domestic rabbits. The oral cavity in the area of the diastema is also affected by skull shape changes. In dorsoventral direction, it is compressed in wild rabbits (negative RW 2 scores), whereas it is expanded in domestic rabbits (positive RW 2 scores) (Figure 2C).

### 3.5. Covariance

**Figure 5:**
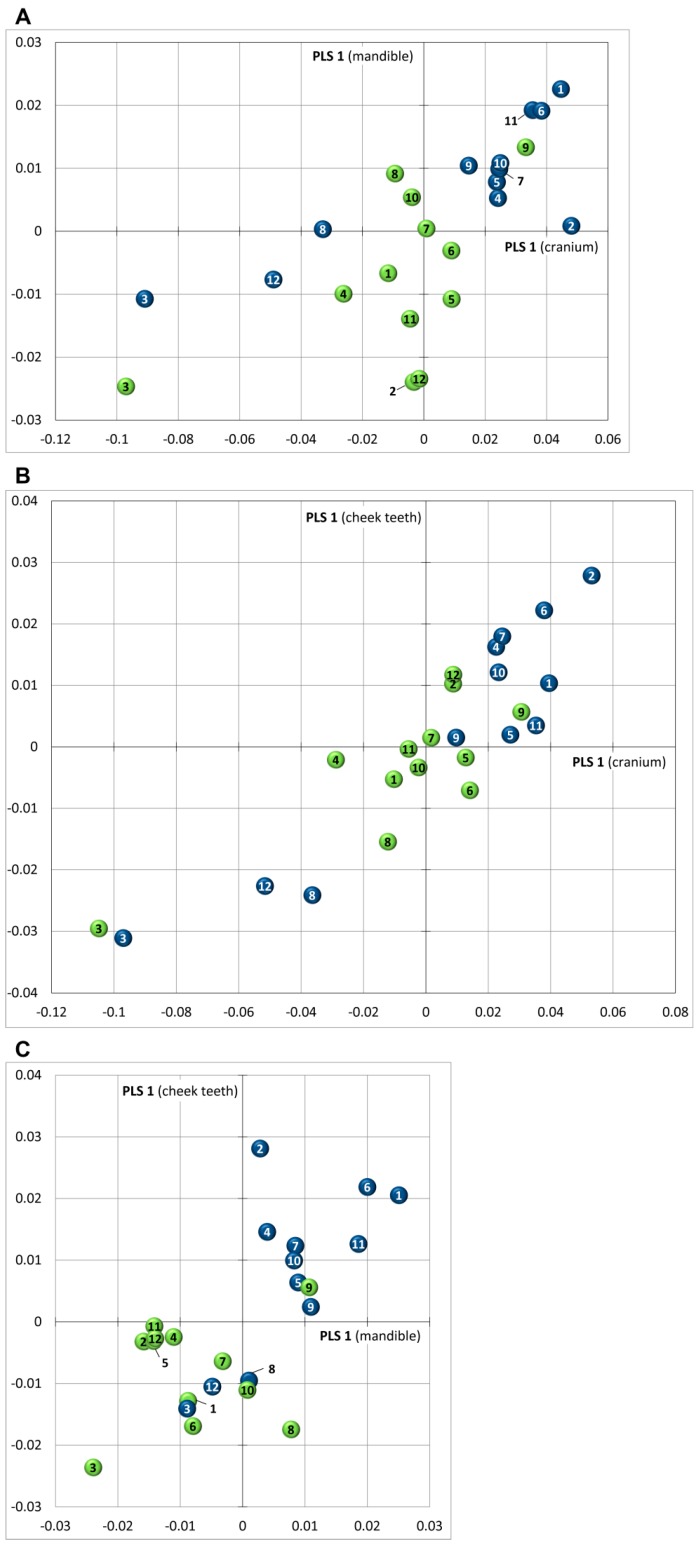
Two-block partial least squares (2-block PLS) analysis testing modular covariation between (**A**) cranium and mandible; (**B**) cranium and cheek teeth; (**C**) mandible and cheek teeth. Color coding as in Figure 2 and Figure 4.

PLS 1 explains between 60% and 90% of the covariance between the three modules (Table 5). Testing the associations between two blocks of variables reveals a strong covariance between module 1 (cranium) and module 2 (mandible) for wild rabbits, whereas no correlation is detected for domestic rabbits (Figure 5A, Table 6). For both groups, the relationship between module 1 (cranium) and module 3 (cheek teeth) is significant (Figure 5B, Table 6). There is a weak covariance between module 2 (mandible) and module 3 (cheek teeth) in wild rabbits, and no correlation was detected for domestic rabbits (Figure 5C, Table 6).

## 4. Discussion

### 4.1. Morphological Diversification and Allometry

Evolution of craniomandibular shape in rabbits has been governed by ecological adaptation [56] including locomotion [57] and dietary habits [58,59]. Our analyses show that there are consistent differences in skull shape between wild and domestic rabbits. We find little overlap of the groups in the RWA (Figure 2A) and complete separation as revealed by the discriminant analysis (Figure 3). These results indicate that the craniomandibular system in wild and domestic rabbits was subjected to different constraints generating phenotypic divergence. The shape variation between both groups is partly coupled with skull size, and morphological differences are therefore partially the result of allometry. A future study including a greater variety of domestic rabbits (larger and smaller specimens, different breeds) may help to clarify the influence of morphological variation with changing size. Allometry is a major factor in the diversification of many domestic mammal breeds and, thus, may also be important in pet rabbits. Nevertheless, our study quantifies the observation that human-imposed artificial selection has led to non-adaptive variation in skull morphology in domestic rabbits [12].

### 4.2. Variance and Covariance in Skull Shape: Implications for Diagnostic Analysis (Clinical Relevance)

**Figure 6:**
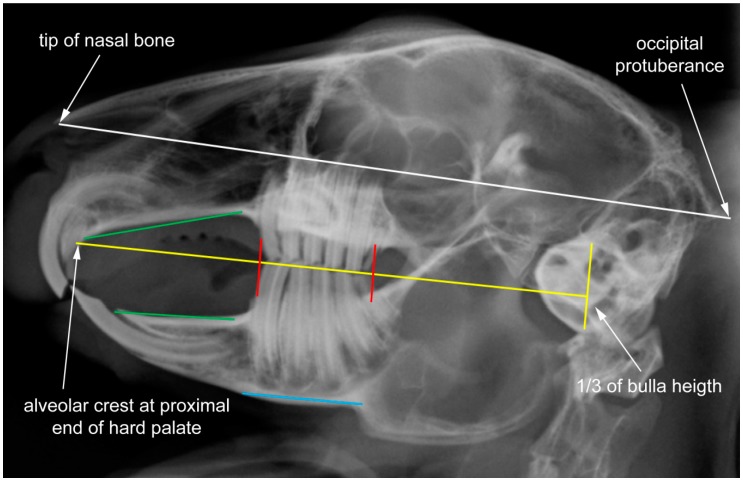
Species-specific reference lines superimposed on the radiograph of a clinically healthy pet rabbit in laterolateral view (according to [4]). The radiographic anatomic reference lines enable objective interpretation of malocclusion in domestic rabbits.

In accordance with the breeding for “cuteness” (concept of baby schema, “Kindchenschema”), the present analysis reveals that the skull shape is generally more quadratic in domestic rabbits, whereas wild rabbits tend to have a long and flat skull. In particular, the relative length of the nasal bone (represented by LM 1) and the occiput (represented by LM 14) characterize this difference. In domestic rabbits, the reference line that marks the dorsal limitation of the maxillary tooth apices in lateral view of the skull is defined to connect the most anterior point of the nasal bone with the most posterior point of the occipital protuberance (white line in Figure 6) [4]. However, the application of this reference line in most wild rabbits might mistakenly indicate retrograde apical elongation of the maxillary cheek teeth—depending on the individual skull shape. Therefore, it is recommended that the non-modified application of this line is primarily restricted to pet rabbits.

### 4.3. Effect of Variation in Incisor Region

Interestingly, the dentition itself forms a relatively unalterable unit that appears not to be essentially affected by the breeding for a shorter skull (“cuteness”) or the evolution towards a shorter skull. This is based on the fact that despite the significant difference in the shape of the cranium between wild and domestic rabbits, the morphological configuration of the teeth themselves (represented by LM 2–11) is very similar across all samples. However, a closer look at the three sub-units of the dentition (incisors, diastema and molars) reveals that the tip of the maxillary incisors tends to project more ventrally in relation to the tip of the mandibular incisors in wild rabbits. This indicates slightly longer clinical crowns of the maxillary incisors. In contrast, the maxillary and mandibular incisors in domestic rabbits tend to occlude more bluntly with a slightly less chisel-shaped tooth tip. This may increase predisposition to incisor malocclusions with subsequent cheek tooth overgrowth.

Normally, incisors and cheek teeth are kept in shape by the continual processes of attrition and abrasion, respectively, which are compensated by continuous growth of the teeth (hypselodont teeth). Accordingly, in rabbits with a healthy dentition, an eruption rate of approximately 2.0 mm per week was recorded in the maxillary incisors and 2.4 mm per week in the mandibular incisors [60,61]. The persistent wear of the hypselodont teeth is basically induced by the natural fibrous diet of rabbits which is very abrasive due to the presence of lignin, cellulose and hard silicate phytoliths in grasses and other plants. Free living rabbits also strip bark off trees with their incisors and chew it just as they ribble at delicate roots. In addition to that, animals with a healthy dentition grind their incisors and cheek teeth periodically which is called “thegosis” or “bruxism”. These planning jaw movements occur in the absence of food and help to maintain a physiological length and shape of the teeth. Thegosis is seen predominantly when rabbits are at rest [62]. Rabbits with a malocclusion, however, often avoid these special jaw movements due to dental pain. Elongated clinical crowns of both the incisors and cheek teeth are a consequence of this.

In rabbits with a healthy dentition, the incisors are continuously worn down during the biting and chewing of each masticatory cycle [63]. While rabbits graze longer grasses, the relatively resistant stems are taken into the mouth and cut near the ground between the incisors [64,65]. Hereby, the incisors meet edge to edge and then the mandibular incisors slide along the caudal surface of the maxillary first incisor, in a predominantly sagittal direction [63]. This reduces the food to manageable pieces that are transported by the tongue to the cheek teeth for further reduction. Pet rabbits fed predominantly on pelleted diets and chopped hay miss this action which might promote a blunter shape and a greater length of the clinical crowns as indicated by the present study.

In addition to that, incisors are worn down continuously while pieces of food are ground between the cheek teeth, provided the food is suitable for physiological jaw movements with a rostrolingually oriented shearing power stroke. This kind of jaw movement is most pronounced in hay mastication and causes that the tips of the mandibular incisors move forward till they touch the dorsal edge of the wear facet of the maxillary incisors (circular upward motion). Furthermore, they are swept transversely across the caudal aspect of the second maxillary incisors [64]. However, when the cheek teeth are crushing (carrot mastication), the mandibular incisors move merely upwards, in a predominantly vertical direction while their tips remain just caudal to the wear facets of the maxillary incisors [64,66]. This might be an additional explanation for the blunter occlusal plane of the maxillary incisors in the pet rabbit group in contrast to the more pointed incisor tip in the wild rabbit group.

### 4.4. Constraints in Molar Region

The present landmark analysis shows that the area lying between the maxillary and mandibular diastema (represented by LM 2, 4–7 and 11) is relatively long and flat in wild rabbits, whereas it is distinctly shorter and higher in domestic rabbits. In pet rabbits with a healthy dentition, the reference lines that mark the inclination of the palatine and mandibular bone plate slightly converge rostrally (green lines in Figure 6) [4]. This is also true for wild rabbits, but the amount of convergence is in general lower than in pet rabbits due to their slightly shorter cheek teeth and longer skulls. This is based on the fact that the clinical crowns of the cheek teeth (represented by LM 6–11) are moderately shorter in wild rabbits compared to those in domestic rabbits with their higher skulls (Figure 2C). This coincides with a study on chinchillas that showed the cheek teeth in pet animals being generally longer in axial direction compared to their wild counterparts [67]. However, the present work demonstrates that the molar sub-unit of the dentition reveals almost no variance in its morphological configuration to other skull structures, both in wild and domestic rabbits. This suggests that the morphofunctionality of the cheek teeth as a unit seems not to be essentially influenced by the domestication process. Yet, analyzed more in detail, RW 2 reveals a tendency of the cheek teeth to shift caudally in domestic rabbits, whereas in the longer skulls of wild rabbits a more rostral shift seems to dominate (Figure 2C).

### 4.5. Implications of Craniomandibular Shape Variation for Masticatory Performance

Another difference between domestic and wild rabbits concerns the position of the most posterior dorsal point of the angular process (represented by LM 13). The area which is defined by the antegonial notch of the mandibular ramus, the angular process and the posterior intersection between mandible and last mandibular molar (m3) (represented by LM 12, 13 and 9) forms a nearly right-angled triangle both in wild and domestic rabbits. In the latter, however, the mandibular ramus is posteriorly higher according to the relative position of the angular process which is gently shifted dorsally (Figure 2C). This results in a decrease of the distance between the jaw articulation and the muscle insertion near the angular process in pet rabbits compared to their wild counterparts. The difference in this distance may have an effect on important jaw closers, such as the posterior deep masseter and the medial pterygoid [66]. The force producing capacity of these muscles (in particular the medial pterygoid) is very high [59] and, thus, differences in the anatomical arrangement of the muscles potentially could be expected to influence bite force. However, future studies measuring the bite force and investigating the muscular differences between domestic rabbits and wild rabbits are necessary.

In this context, it is interesting to note that the part of the mandible that lies ventrocaudal to the antegonial notch (reaching up to the most posterior dorsal point of the angular process) is more pronounced in domestic rabbits than in their wild counterparts (Figure 1A). This may be a normal consequence of the progressive increase in skull height or it indicates the presence of stronger jaw muscles since this part of the mandible represents the major attachment area for the superficial and anterior deep part of the masseter muscle both acting as jaw closer. The latter statement seems more realistic since the muscle attachment areas seem to be more salient in pet rabbits (Figure 1A). In addition to that, the present landmark analysis reveals that the area depicting the superficial masseter (represented by LM 11–13) is noticeably larger in pet rabbits compared to data found in wild rabbits (Figure 2C). On one hand, the muscle needs space for its attachment onto the bone, but on the other hand it may also influence the shape of the mandible due to the forces it exerts [68]. Further studies focusing on muscular anatomy in more detail are needed to verify if the masseter muscle fibers are more vertically aligned in pet rabbits due to the relative shortness of the skull. In positive terms, it might be possible to assume that on basis of the correlation between bone shape and muscle properties, both facts probably influence the bite force at the cheek teeth area which then should be larger in domestic rabbits compared to the wild animals with their longer skulls.

It is generally assumed that in rabbits, dietary habits seem to be a major factor in developing acquired malocclusions (reviewed in [1]). Although the dentitions of the wild and domesticated rabbit seem to be in principle identical (confirmed by the present study), their diet definitely differs. Wild animals commonly eat lush green grasses, young tree shoots and delicate roots, while pet rabbits mostly consume a primarily pellet-type diet with additionally offered hay (freely available) which is more resistant than grass. This basic diet is especially popular among most rabbit breeders. Pet owners like to complement or replace this diet in part with a certain amount of daily offered fresh leafy and root vegetables. Furthermore, small pieces of fruits are given as treats. Since diet is known to largely influence skull morphology of different vertebrates [69] (Table 7), it is important to look first at the basic jaw movements in chewing rabbits which considerably differ dependent on the food resistance and are accompanied by a varying degree of strain on the incisors and cheek teeth.

Each masticatory cycle consists of a biting (see above) and chewing sequence [63]. Chewing starts with a jaw opening phase that is followed by a fast closing of the jaw. Subsequently, food is ground or crushed between the cheek teeth unilaterally during the slow closing phase of the masticatory cycle [66]. While the basic chewing rhythm is not affected by the food texture [65], the jaw movement, however, strongly depends on the type of food that is ingested (shearing or crushing power stroke) [63,66]. In addition to that, the force that is applied by the cheek teeth during crushing increases in proportion to the hardness of the food [63].

During the shearing stroke which is primarily used in hay mastication the working side condyle moves forward from a strongly retracted position while the balancing condyle shifts slightly backward. Consequently, the mandibular cheek teeth on the working side are moved lingually (3–4 mm) and slightly rostrally (1 mm) with minimum vertical and maximum transverse jaw excursion [66]. Thus, they perform a buccolingually directed power stroke where a considerable shearing force is applied between the interlocked transverse ridges of the upper and lower (pre-) molars.

This jaw movement sometimes occurs in pellet mastication, but is never seen in carrot mastication. Carrots are always chewed with the aid of a crushing stroke where the position of the working condyle is initially more anterior. The forward movement of this condyle is less pronounced while the backward movement of the balancing condyle is enhanced. The mandibular cheek teeth of the working side move purely lingually without a rostromedial shearing action, just swinging slightly upward in the buccal and swinging slightly downward in the lingual phase. There is a maximum vertical gape and the result is primarily a crushing action that can also be observed in rabbits eating pellets. However, this type of chewing is never seen in hay mastication.

In summary, this suggests that in grazing wild rabbits cheek teeth are strained primarily in a laterorostral direction while shear forces on the interlocked enamel crests dominate and there is only a small amount of axial load on the cheek teeth. In this context, it is reasonable that the first mandibular cheek tooth is the largest of the rabbit dentition. Thus, the teeth lying behind it can firmly prop up against this stronger premolar. In contrast to wild animals, most pet and breeding rabbits predominantly crush “unnatural” food between their teeth (pellets, carrots and other root vegetables) which is accompanied with a much higher axial strain on the (pre-)molars and an insufficient tooth wear (higher clinical crowns) combined with a tendency to retrograde tooth elongation [1]. This fact appears also to explain why longitudinal splits of the first mandibular premolar (P3) are so common in pet rabbits. They are assumed to be the consequence of a load-related apical irritation that results in an abnormal tooth tissue formation (hypoplasia). Thus, the altered cement fails to connect both tooth bodies firmly together (bilophodont cheek teeth) resulting in a longitudinally “split” tooth [1].

Considering additionally that hay is more resistant than fresh grasses, it seems logical to develop further the hypothesis that pet and breeding rabbits had to develope stronger jaw muscles and secondarily larger axial bite forces than their wild counterparts to be able to crush their unnatural food more effectively. This might be supported by a shorter skull and more vertically oriented muscle fibers whereas a longer skull with a more anteriorly positioned masseter muscle (as seen in wild rabbits) reduces the vertical bite force due to a greater distribution of bite forces on all cheek teeth. As teeth at the rear of the dentition generally exert higher bite forces than the more rostrally positioned teeth, this might be an explanation for the found tendency of the cheek teeth to shift caudally in the group of the domestic rabbits. Furthermore, the presence of stronger muscles may explain the more salient appearance of the caudoventral part of the masseteric fossa (mandibular angle) in pet rabbits, as in different mammals (re-)modeling of the mandibular cortical bone has proven to be associated with oral processing of tough food (reviewed in [70]). This research has shown that especially a postnatal variation in diet-related jaw-loading patterns had a marked influence on the masticatory bone formation, leading to morphological variations between sister taxa in the long term [70]. With age, however, plasticity decreases. Based on this, rabbit breeders feeding predominantly pellets and hay seem to promote malocclusions in adult rabbits unknowingly as the masticatory apparatus of the weanlings is exposed to unphysiological strains that may result in changes of the skull morphology.

### 4.6. Phenotypic Plasticity in the Mammalian Feeding Apparatus

A series of studies have supported the hypothesis that an increase in jaw robustness is an evolutionary or plastic response (phenotypic plasticity) to generating higher-magnitude loads. They all found load-related morphometric variations and phenotypic changes in jaw and skull morphology in many different mammals (rabbits, chinchillas, rats, mice, ferrets, minipigs, lions, tigers, primates) being fed diets of different mechanical properties [12,67,69,70,71,72,73,74,75,76,77,78,79,80,81,82,83,84,85,86,87,88,89,90,91,92,93,94,95,96,97,98,99] (Table 7).

All studies showed a positive correlation between dietary properties and peak masticatory loads that caused the adjacent cortical bony tissue to change its structure and morphology whereby it normally became thicker and more mineralized. Therefore, rabbits and primates that routinely ingested stiff and tough food exhibited relatively larger jaws to counter elevated peak masticatory stresses (peak bite force) (reviewed in [70]). In rabbits, hay and pellets resulted in greater jaw-muscle activity and higher mandibular strain, compared to the ingestion of carrots [80]. Hay seems to be the most mechanically challenging food as it is tougher and stiffer than pellets and carrots [70]. It requires more chews per gram to be processed which results in longer chewing bouts compared to pellets and carrots. This means that over a longer period of time the teeth are predominantly axially loaded due to the elevated bite force. If we take into consideration that hay with a lot of hard stems has reduced nutritive properties and potential limits on digestibility, then rabbits eating predominantly hay need to consume large quantities to meet basic metabolic and nutritional demands [70]. All of this promotes retrograde tooth elongation and incursion of the apices into the adjacent bone (most common finding in malocclusions) [1]. Furthermore, hay also promotes periodontal diseases (impacted food) and, therefore is not the best nutrition for rabbits [31]. Grasses and other fresh plants, however, are abrasive, but relatively soft and, thus, can be ground down with relatively low axial load of the cheek teeth as the primary strain on the (pre-) molars occurs in a more physiological laterorostral direction with the aid of the shearing power stroke.

## 5. Conclusions

The present study is an intriguing example that highlights the importance of integrating evolution and veterinary science in order to improve the knowledge base. Evolutionary Veterinary Science is key to gain a comprehensive understanding of pathologies and, thus, opens up new avenues of research in veterinary medicine.

**Figure 7:**
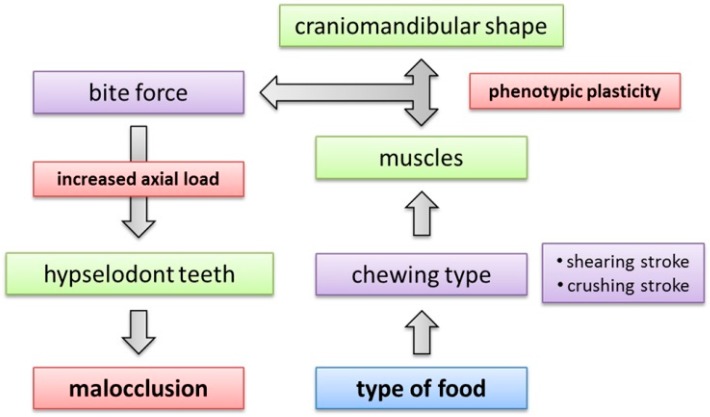
Flowchart summarizing the mechanisms involved in food-masticatory apparatus interactions as indicated by the present study. Type of food is the critical factor because it determines the performed chewing mechanism in rabbits. The chewing mechanism constrains the muscle performance, which has considerable impact on the craniomandibular shape via phenotypic plasticity. The musculoskeletal arrangement influences the bite force that acts on the teeth. The higher the bite force, the greater the axial load increasing the risk of malocclusions.

In summary, the landmark-based geometric morphometric analysis indicates that the craniomandibular shape of rabbits changed at different rates in the course of domestication since cranial morphometry strongly differs between domestic and wild rabbits although the dentition itself does not seem to differ significantly. This leads to a functional imbalance of the masticatory apparatus because the regions that are associated with the generation of masticatory forces (i.e., cranium and mandible) change independently from the regions that are associated with the resistance of masticatory forces (i.e., hypselodont teeth). Finally, this disequilibrium seems to result in a predisposition to dental problems in domestic rabbits. What caused shorter skulls in the course of the domestication? On the one hand, selective breeding for extremely short crania in dwarf rabbits is sometimes accompanied with the occurrence of extremely short skulls (brachygnathic rabbits with a shorter maxillary diastema and secondary congenital incisor malocclusion). On the other hand, it has been proven that diet has a significant influence on skull morphology as well (phenotypic plasticity) (Figure 7).

The present analysis comparing wild and domestic rabbits shows that in pet animals an increased skull height with a concurrently greater muscle insertion area (ventrocaudal enlargement of the mandibular ramus) and more vertically oriented jaw muscle fibers exhibit higher muscle strength and, thus, a larger bite force compared to wild rabbits. Previous studies confirm that the shorter skull morphology seems to be a long-term adaptation to the increased stress on the dentition due to feeding a diet consisting predominantly of harder particles than that found in the wild (pellets, hay, carrots) [70]. Instead of performing lateral gliding jaw movements which grind the hypselodont cheek teeth optimally in the long term, the more resistant food particles (stiff hay, pellets and carrots) are predominantly crushed between the teeth which requires stronger hinge movements (raising and lowering the jaw) [102]. Consequently, the cheek teeth have to withstand a higher masticatory pressure which also causes abnormal stress to the nearby bone with all its consequences (retrograde tooth elongation which is the most common finding in pet rabbits with a beginning or far advanced malocclusion [1]. Based on this knowledge, the diet of pet rabbits has to be strictly reconsidered as this has been already recommended by Böhmer [31]. A more natural nutrition of domestic rabbits appears to be all the more important because the present results show that, even in rabbits with a primarily healthy dentition, all cheek teeth already show an elongated clinical crown which makes the teeth much more susceptible to an abnormal axial load with secondary bending or shifting forces. All these facts strengthen the importance to offer pet rabbits an adequate close-to-nature nutrition throughout the whole life and especially beginning early parallel to weaning (phase of increased phenotypic plasticity) that ensures a normal strain on the teeth by promoting physiological lateral gliding movements and avoiding direct axial load [31].

## Figures and Tables

**Table 1:** Definition of landmarks (LM) (type I and II (sensu [50])) applied to laterolateral radiological images of the skull in wild and domestic rabbits.

LM	Type	Definition
1	II	most anterior point of nasal bone
2	I	intersection between second maxillary incisor (I2) (peg tooth) and maxillary bone
3	II	most anterior tip of first maxillary incisor (I1)
4	II	most anterior tip of first mandibular incisor (i1)
5	I	intersection between first mandibular incisor (i1) and mandibular bone
6	I	anterior intersection between mandible and first mandibular cheek tooth (p2)
7	II	most anterior point of occlusal plane between maxillary and mandibular first cheek tooth (P2, p2)
8	II	most posterior point of occlusal plane between maxillary and mandibular last molar (M3, m3)
9	I	posterior intersection between mandible and last mandibular molar (m3)
10	I	posterior intersection between maxillary bone and last maxillary molar (M3)
11	I	anterior intersection between maxillary bone and first maxillary cheek tooth (P2)
12	II	antegonial notch of mandible
13	II	most posterior dorsal point of angular process
14	II	most posterior point of occipital protuberance

**Table 2:** Variance and cumulative variance percentages per relative warp (RW).

RW	Variance (%)	Cumulative Variance (%)
RW 1	54.60	54.60
RW 2	13.24	67.83
RW 3	9.91	77.74
RW 4	5.25	83.00
RW 5	3.72	86.72
RW 6	2.80	89.52
RW 7	2.67	92.19
RW 8	2.14	94.33
RW 9	1.40	95.73

**Table 3:** Multivariate regression of log centroid size against the first nine relative warps (RW), with coefficient of determination (r²) and significance value (*p*-value) for the null hypothesis. Asterisk (*) marks significant *p*-value.

	Log Centroid Size	
	r²	*p*-Value
RW 1	0.035066	0.38092
RW 2	0.168757	0.046143 *
RW 3	0.010572	0.63258
RW 4	0.510982	8.67 × 10 ^−5^ *
RW 5	0.023031	0.47901
RW 6	0.017082	0.54269
RW 7	0.046522	0.31144
RW 8	0.001987	0.83617
RW 9	0.004693	0.75044

**Table 4:** Variance (s²) at applied landmarks (LM) (sorted in descending order).

LM	Variance (s²)
1	1.6041 × 10 ^−3^
14	0.32197 × 10 ^−3^
12	0.23093 × 10 ^−3^
6	0.20758 × 10 ^−3^
13	0.1961 × 10 ^−3^
2	0.1612 × 10 ^−3^
11	0.13185 × 10 ^−3^
4	0.12278 × 10 ^−3^
5	0.10884 × 10 ^−3^
7	0.10744 × 10 ^−3^
9	0.10476 × 10 ^−3^
10	0.09878 × 10 ^−3^
8	0.08468 × 10 ^−3^
3	0.07512 × 10 ^−3^

**Table 5:** Covariance and cumulative covariance percentages per partial least squares axis (PLS).

PLS	Covariance (%)	Cumulative Covariance (%)
module 1 vs. 2		
PLS 1	82.08	82.08
PLS 2	12.35	94.43
module 1 vs. 3		
PLS 1	90.53	90.53
PLS 2	8.51	99.04
module 2 vs. 3		
PLS 1	59.55	59.55
PLS 2	22.60	82.15

**Table 6:** Linear regression of partial least square axis (PLS) 1 of module 1 (cranium) vs. 2 (mandible), 1 vs. 3 (cheek teeth) and 2 vs. 3, with coefficient of determination (r²) and significance value (*p*-value) for the null hypothesis. Asterisk (*) marks significant *p*-value.

Module	Log Centroid Size	
	r²	*p*-Value
module 1 vs. 2 (wild rabbits)	0.66167	0.0012895 *
module 1 vs. 2 (domestic rabbits)	0.27903	0.077491 *
module 1 vs. 3 (wild rabbits)	0.85269	1.82 × 10 ^−5^ *
module 1 vs. 3 (domestic rabbits)	0.64699	0.0016086 *
module 2 vs. 3 (wild rabbits)	0.46661	0.014344 *
module 2 vs. 3 (domestic rabbits)	0.034312	0.56437

**Table 7:** Influence of food on skull morphology, muscle anatomy and tooth length (phenotypic plasticity). Abbreviations: TMJ = temporomandibular joint, ref. = reference.

Species	Ref.	Diet Fed	Feeding Period	Background	Results (Morphology, Anatomy)
laboratory mice (3 weeks old)	[88]	rodent pellets vs. ground pellets mixed with jelly	about 5 months	food consistency significantly influenced bone remodeling (shape of the mandible) as hard food generates greater stress in the jaw (bone remodeling)	mice fed on hard food displayed mandibles functionally more efficient for hard-food processing (higher mechanical advantage values), extended coronoid and angular processes, ventrally expanded incisor and molar zones; all functional modules except the molar zone showed shape differences. Mice fed on soft food showed jaw elongations (reduced mechanical advantage values)
mice (after weaning), healthy animals and mice with muscle dystrophy (pathological muscular defect)	[87]	hard pellets vs. pellets under the form of jelly	30 weeks	remodeling of the mandible as response to food consistency and muscular dystrophy	significant changes in mandible size whereby some parts of the mandible were more prone to remodeling (such as the angular process which is less robust when fed soft diet)
rats	[83]	hard diet vs. soft diet	about 4 months	in particular, the mandible depends on muscular function to grow to its normal size, maxillary growth seems to be under closer genetic programming	soft-diet animals had smaller jaw muscles and smaller jaws
farm-reared long-tailed chinchillas vs. museum skulls	[73]	granular feed (pellets) vs. natural diet	life-long	under natural habitat conditions, fiber constitutes almost 66% of the chinchilla diet, whereas under conditions of farm and domestic keeping granular feed with the fiber ranging from 12% to 18% is the main food; this does not require such hard work of the masticatory apparatus	crania and mandibles of farm-reared chinchillas were significantly larger than the museum specimens; only the frontal length did not show any significant differences between both groups; the length of the maxillary cheek-tooth row was larger in the museum crania
domestic (captive-bred) long-tailed chinchillas vs. wild-caught chinchillas and zoo specimens	[67]	granular feed (pellets) vs. natural diet	lifelong	captive bred animals with a normocclusion had longer cheek teeth (7.4 mm) than wild-caught chinchillas (5.9 mm) due to prolonged chewing of the naturally abrasive diet, zoo specimens lay in between (6.6 mm)	skulls of captive-bred chinchillas were on average 16% longer and slightly higher than the others (assumed to unrestricted food intake)
suckling rabbits	[100]	small food particles vs. milk	about 4 weeks	postnatal development of the masticatory apparatus due to change in function from suckling to chewing (shift of muscle activity)	the facial skull becomes higher and longer, increase in mandibular height and development of an angular process, anterior part of the superficial masseter attains a more vertical position, displacement of the mandibular angle in a ventroposterior direction, stronger jaw closing muscles and increased bite-force
juvenile rabbits	[99]	hard pellets vs. soft pellets (soaked in water)	87 days	influence of food consistency on the rabbit masseter muscle fibers (plasticity)	rabbits adjusted to altered foods within days resulting in changes in the masseter muscle; hard-diet animals increased the occlusal forces (larger fiber cross-sectional area); soft-diet animals decreased the occlusal forces (small fiber cross-sectional area)
rabbits (weanlings)	[74]	soft and hard/tough diet	15 weeks	influence of masticatory stresses on the development and structure of the hard palate (phenotypic plasticity)	rabbits subjected to elevated masticatory loading developed hard palates with significantly greater bone area, greater cortical bone thickness and thicker anterior plates
rabbits (weanlings)	[89]	ground rabbit pellets vs. intact pellets and hay blocks	105 days	phenotypic plasticity of the superficial masseter fiber architecture as dietary consistency influences its fiber type composition	tough diet causes an increase in physiological cross-sectional areas of the masseter muscle (increased muscle mass)
New Zealand rabbits (weanlings)	[101]	powdered pellets, intact pellets, intact pellets and hay blocks	26 weeks	diet-induced variations in masticatory stresses influence postorbital soft tissues (fibrocartilage)	more degraded organization of collagen fibers in the postorbital region due to increased masticatory forces (pellets and hay)
New Zealand white rabbits (4-week-old weanlings)	[82]	ground pellets vs. intact pellets with hay blocks	15 weeks	diet-related variation in masticatory stress affects structural properties and extracellular matrix composition of the TMJ and the symphysis (histology and immunohistochemistry of articular cartilage revealed a diminished articular cartilage viscoelasticity)	elevated masticatory loads result in an increase of the masseter muscle mass and a partial skull bone enlargement (mandibular corpus, condyle, symphysis) with a greater local bone density
ferrets (5 weeks old)	[84]	hard pellets vs. soft pellets (soaked in water)	6 months	effect of masticatory muscle function on craniofacial morphology	less tension on the periosteal membrane of the cranial bones, resulting in less periosteal bone apposition in the inserting areas

## Supplementary Materials

The following are available online at www.mdpi.com/2306-7381/4/1/5/s1, Table S1: 2D landmark coordinates of the analyzed specimens.
