# Supplementary material for: Shape Variation in the Craniomandibular System and Prevalence of Dental Problems in Domestic Rabbits: A Case Study in Evolutionary Veterinary Science
Source: Vet Sci. 2017 Jan 24;4(1):5. doi: 10.3390/vetsci4010005 (PMC5606619; doi:10.3390/vetsci4010005)
Supplement: Supplementary file 1 [file vetsci-04-00005-s001.pdf]

Supplementary Materials: Shape Variation in the Craniomandibular System and Prevalence of Dental Problems in Domestic Rabbits: A Case Study in Evolutionary Veterinary Science

Christine Böhmer *, Estella Böhmer

Table S1: Collected 2D landmark coordinates (LM 1–14) of the analyzed specimens. Abbreviations: ID = identification number, W = wild rabbit, D = domestic rabbit.

Specimen	x-Coordinate	y-Coordinate
ID = W1	85.0	385.0
	118.0	250.0
	101.0	199.0
	123.0	214.0
	176.0	199.0
	343.0	226.0
	357.0	275.0
	473.0	281.0
	477.0	260.0
	473.0	317.0
	338.0	323.0
	492.0	124.0
	740.0	211.0
	918.0	359.0
ID = W2	36.0	382.0
	93.0	197.0
	69.0	153.0
	91.0	162.0
	148.0	150.0
	344.0	190.0
	363.0	259.0
	497.0	255.0
	505.0	232.0
	512.0	289.0
	346.0	313.0
	509.0	63.0
	778.0	187.0
	959.0	317.0
ID = W3	172.0	452.0
	86.0	203.0
	65.0	159.0
	86.0	169.0
	140.0	152.0

	317.0	163.0
	335.0	239.0
	474.0	243.0
	481.0	228.0
	489.0	285.0
	305.0	307.0
	502.0	59.0
	774.0	183.0
	967.0	359.0
**ID = W4**	62.0	395.0
	94.0	222.0
	69.0	177.0
	86.0	185.0
	150.0	164.0
	341.0	192.0
	357.0	256.0
	493.0	254.0
	501.0	232.0
	508.0	281.0
	337.0	306.0
	513.0	54.0
	764.0	187.0
	964.0	362.0
**ID = W5**	79.0	475.0
	87.0	295.0
	64.0	235.0
	87.0	243.0
	152.0	216.0
	322.0	215.0
	355.0	285.0
	476.0	265.0
	480.0	240.0
	498.0	292.0
	340.0	350.0
	492.0	71.0
	747.0	153.0
	959.0	286.0
**ID = W6**	36.0	311.0
	88.0	154.0
	80.0	109.0
	94.0	126.0
	163.0	119.0
	338.0	169.0
	353.0	223.0
	473.0	231.0

	481.0	217.0
	475.0	265.0
	320.0	271.0
	493.0	72.0
	758.0	211.0
	900.0	386.0
**ID = W7**	59.0	488.0
	73.0	302.0
	44.0	259.0
	63.0	265.0
	134.0	235.0
	325.0	237.0
	347.0	305.0
	479.0	282.0
	486.0	261.0
	496.0	307.0
	330.0	362.0
	467.0	91.0
	727.0	187.0
	964.0	337.0
**ID = W8**	118.0	515.0
	66.0	298.0
	37.0	251.0
	59.0	265.0
	109.0	235.0
	290.0	215.0
	316.0	288.0
	450.0	273.0
	457.0	254.0
	472.0	304.0
	294.0	362.0
	454.0	82.0
	743.0	161.0
	968.0	296.0
**ID = W9**	65.0	439.0
	79.0	258.0
	45.0	209.0
	67.0	213.0
	121.0	199.0
	305.0	203.0
	340.0	280.0
	479.0	267.0
	482.0	242.0
	500.0	293.0
	320.0	335.0

	473.0	76.0
	754.0	169.0
	971.0	342.0
**ID = W10**	63.0	432.0
	93.0	254.0
	64.0	209.0
	84.0	217.0
	148.0	197.0
	338.0	209.0
	355.0	274.0
	491.0	267.0
	498.0	242.0
	507.0	300.0
	329.0	326.0
	493.0	69.0
	750.0	187.0
	967.0	355.0
**ID = W11**	60.0	464.0
	86.0	288.0
	63.0	238.0
	89.0	244.0
	156.0	228.0
	323.0	233.0
	345.0	301.0
	494.0	281.0
	501.0	269.0
	506.0	303.0
	326.0	350.0
	495.0	91.0
	775.0	182.0
**ID = W12**	116.0	482.0
	65.0	251.0
	43.0	203.0
	65.0	214.0
	120.0	194.0
	296.0	197.0
	323.0	276.0
	463.0	272.0
	473.0	254.0
	483.0	310.0
	301.0	342.0
	500.0	77.0
	759.0	196.0
	970.0	357.0
**ID = D1**	108.0	381.0

	121.0	285.0
	114.0	255.0
	129.0	247.0
	150.0	251.0
	235.0	262.0
	246.0	298.0
	301.0	302.0
	310.0	285.0
	320.0	322.0
	231.0	330.0
	309.0	217.0
	439.0	286.0
	539.0	371.0
**ID = D2**	189.0	791.0
	153.0	500.0
	111.0	440.0
	134.0	412.0
	206.0	357.0
	499.0	338.0
	534.0	425.0
	723.0	394.0
	742.0	351.0
	755.0	438.0
	523.0	518.0
	714.0	159.0
	1083.0	266.0
	1399.0	387.0
**ID = D3**	365.0	798.0
	149.0	540.0
	96.0	457.0
	117.0	455.0
	192.0	410.0
	436.0	358.0
	499.0	467.0
	689.0	401.0
	683.0	362.0
	715.0	446.0
	492.0	551.0
	640.0	139.0
	1064.0	232.0
	1403.0	393.0
**ID = D4**	180.0	682.0
	123.0	404.0
	81.0	328.0
	106.0	339.0

	218.0	304.0
	465.0	314.0
	512.0	414.0
	723.0	389.0
	728.0	363.0
	740.0	444.0
	492.0	494.0
	724.0	104.0
	1121.0	303.0
	1418.0	523.0
**ID = D5**	96.0	559.0
	82.0	381.0
	38.0	343.0
	50.0	332.0
	98.0	289.0
	282.0	253.0
	311.0	316.0
	449.0	272.0
	443.0	257.0
	463.0	305.0
	307.0	379.0
	396.0	90.0
	692.0	151.0
	911.0	249.0
**ID = D6**	140.0	725.0
	188.0	477.0
	111.0	375.0
	143.0	383.0
	230.0	336.0
	449.0	322.0
	508.0	430.0
	716.0	381.0
	728.0	349.0
	742.0	441.0
	505.0	503.0
	688.0	116.0
	1099.0	271.0
	1371.0	479.0
**ID = D7**	318.0	1047.0
	214.0	780.0
	126.0	713.0
	150.0	722.0
	230.0	643.0
	490.0	517.0
	581.0	607.0

	762.0	493.0
	752.0	450.0
	802.0	530.0
	594.0	709.0
	611.0	229.0
	1110.0	202.0
	1478.0	318.0
**ID = D8**	194.0	826.0
	136.0	549.0
	88.0	486.0
	119.0	498.0
	202.0	439.0
	454.0	376.0
	514.0	481.0
	716.0	424.0
	727.0	395.0
	750.0	483.0
	500.0	586.0
	691.0	152.0
	1117.0	253.0
	1462.0	466.0
**ID = D9**	89.0	402.0
	90.0	283.0
	70.0	239.0
	85.0	242.0
	114.0	226.0
	244.0	206.0
	276.0	245.0
	366.0	230.0
	366.0	207.0
	384.0	250.0
	269.0	286.0
	333.0	96.0
	547.0	125.0
	720.0	209.0
**ID = D10**	188.0	774.0
	194.0	489.0
	139.0	408.0
	160.0	420.0
	233.0	355.0
	510.0	337.0
	554.0	450.0
	765.0	416.0
	776.0	383.0
	817.0	470.0

	538.0	550.0
	728.0	129.0
	1168.0	247.0
	1465.0	513.0
**ID=D11**	93.0	391.0
	67.0	255.0
	49.0	214.0
	63.0	212.0
	94.0	195.0
	235.0	178.0
	261.0	229.0
	354.0	213.0
	358.0	193.0
	366.0	234.0
	262.0	277.0
	345.0	66.0
	546.0	133.0
	726.0	211.0
**ID = D12**	185.0	789.0
	158.0	502.0
	108.0	439.0
	128.0	413.0
	197.0	367.0
	494.0	346.0
	529.0	427.0
	720.0	401.0
	725.0	353.0
	760.0	444.0
	527.0	518.0
	712.0	167.0
	1082.0	268.0
	1400.0	394.0

© 2017 by the authors; licensee MDPI, Basel, Switzerland. This article is an open access article distributed under the terms and conditions of the Creative Commons by Attribution (CC-BY) license (http://creativecommons.org/licenses/by/4.0/).
